# Comparative study on muscle function in two different streptozotocin-induced diabetic models

**DOI:** 10.1007/s00592-024-02311-3

**Published:** 2024-06-10

**Authors:** Rahmawati Aisyah, Mion Kamesawa, Mayu Horii, Daiki Watanabe, Yuki Yoshida, Kenshu Miyata, Thanutchaporn Kumrungsee, Masanobu Wada, Noriyuki Yanaka

**Affiliations:** 1 Graduate School of Integrated Sciences for Life, Hiroshima University, Higashi-Hiroshima, 739-8528 Japan; 2 Graduate School of Humanities and Social Sciences, Hiroshima University, Hiroshima, 739-8521 Japan; 3 Graduate School of Sport and Health Sciences, Osaka University of Health and Sport Sciences, Osaka, 564-8565 Japan

**Keywords:** Streptozotocin, Muscle contraction force, Fatigue resistance, Mitochondrial dysfunction

## Abstract

**Aims:**

Streptozotocin (STZ) is widely used to study diabetic complications. Owing to the nonspecific cytotoxicity of high-dose STZ, alternative models using moderate-dose or a combination of low-dose STZ and a high-fat diet have been established. This study aimed to investigate the effects of these models on muscle function.

**Methods:**

The muscle function of two STZ models using moderate-dose STZ (100 mg/kg, twice) and a combination of low-dose STZ and high-fat diet (50 mg/kg for 5 consecutive days + 45% high-fat diet) were examined using in vivo electrical stimulation. Biochemical and gene expression analysis were conducted on the skeletal muscles of the models immediately after the stimulation.

**Results:**

The contractile force did not differ significantly between the models compared to respective controls. However, the moderate-dose STZ model showed more severe fatigue and blunted exercise-induced glycogen degradation possibly thorough a downregulation of oxidative phosphorylation- and vasculature development-related genes expression.

**Conclusions:**

Moderate-dose STZ model is suitable for fatigability assessment in diabetes and careful understanding on the molecular signatures of each model is necessary to guide the selection of suitable models to study diabetic myopathy.

**Supplementary Information:**

The online version contains supplementary material available at 10.1007/s00592-024-02311-3.

## Introduction

In recent decades, diabetes has become a major cause of mortality globally. Multiple complications of diabetes, including cardiovascular disease, neural damage, nephropathy, and retinopathy, are the leading causes of mortality and morbidity among individuals with diabetes [1]. Muscle impairment is a common diabetic complication associated with impaired mobility and disability [2]. As functional skeletal muscle is necessary to slow disease progression, understanding the pathophysiology and molecular mechanisms of diabetic myopathy will help develop better therapeutic strategies and subsequent management of other diabetic complications [3]. Hence, it is important to establish a robust model that represents the observed pathophysiology of patients.

Several studies have evaluated the muscle function, particularly contraction force, in animal models of diabetes [4–7]. Streptozotocin (STZ) is a well-known agent used to generate animal models of diabetes and is commonly used in studies of diabetic complications, including diabetic myopathy [8]. However, owing to its nonspecific cytotoxicity at high doses, it is difficult to dismiss the STZ-induced tissue dysfunction in diabetic mice [9, 10]. In particular, the kidneys have been shown to be susceptible to STZ toxicity via glucose transporter GLUT2 [11]; therefore, the pathological relationship between the kidneys and skeletal muscles after the administration of a high-dose STZ cannot be omitted. Thus, to diminish the nonspecific toxicity of STZ, alternative models employing moderate doses or combining low doses of STZ with a high-fat diet (HFD) have been established [12–14]. Although both models are used interchangeably, how these two models exert effects on muscle function is yet to be confirmed.

Various experimental methods have been used to assess the muscle function in animal models [15–17]. Noninvasive in vivo methods, such as the four-limb hanging and grip strength tests for muscle strength, and treadmill and swimming pool tests for exercise capacity, have been frequently adopted to study muscle disease and treatment effects. Such techniques offer the advantage that testing does not require muscle excision or animal termination, and allows for continuous observation of functional performance. However, these tests often produce highly variable data owing to the individual differences in mouse behavior. Forced tasks can also induce stress in mice, which may mask the actual effects of the disease or treatment [17]. To address this concern, alternative in vivo assays using electrical stimulation of intact muscles of living animals have been developed [16, 18]. This method has been used in multiple studies and has shown high-throughput and physiologically relevant results [18–21]. However, to the best of our knowledge, this method has not been used to assess muscle function in diabetic models.

To date, there have been mixed results of skeletal muscle function in STZ-treated mice, with the majority showing decreased contractile force [5, 7, 22–24]; whereas others observed no change [24–26] or increased contractile force [6, 27, 28], likely due to the variabilities in diabetic models and assessment of muscle function. Furthermore, majority of these studies employed high-dose STZ. Therefore, we investigated whether two alternative STZ models, moderate-dose and low-dose STZ combined with HFD, affect muscle function differently, using in vivo electrical stimulation. Our findings showed a distinct difference in muscle function between the models, likely because of different molecular cues. This suggests that careful consideration of the molecular signatures of each model is necessary to select a suitable model for studying diabetic myopathy.

## Materials and methods

### Animals

All animal experiments were conducted in accordance with the animal care protocol approved by the Animal Use Committee of the Hiroshima University (Ethical approval No. C22-30). Two types of diabetes modes using male ICR mice aged 8-week-old (Charles River Japan) were used in this study. ICR mouse was preferred over C57BL/6 because it is reliably sensitive to the toxin and more susceptible to developing diabetic complications [8, 29, 30]. In the first model, mice fed with commercial chow diet (MF, Oriental Yeast Co., Ltd.) were given moderate-dose STZ (Sigma-Aldrich) (100 mg/kg in 0.1 M citrate buffer, pH 4.5) twice with one day break in between for the test group (MSTZ). Placebo injection (0.1 M citrate buffer, pH 4.5 M) and same chow diet were given to the control group (CON). Mice were analyzed 4 weeks post STZ injections. In the second model, the test group (LSTZ/HFD) was given high-fat diet (HFD) (45% fat, 20, 5% protein, and 34, 8% carbohydrate; Oriental Yeast Co., Ltd) for 4 weeks prior to low-dose STZ injection (50 mg/kg for 5 consecutive days), then maintained on HFD for additional 8 weeks post STZ treatment. The control group (CON) was given commercial chow diet and placebo injection for 5 consecutive days (Fig. S1). Only animals with fasting blood glucose levels > 300 mg/dl were considered diabetic in both models and at least five mice were used for each group. All animals survived until the end of experiments. Mice were housed at 24–26 °C in a 12 h light–dark cycle (8:00–20.00 light cycle, 20.00–8.00 dark cycle) and given water ad libitum.

### Contractile force and fatigability test

In vivo electrical stimulation using surface electrode on the gastrocnemius (GAS) muscle from the left hindlimb was used to examine contractile force and fatigability. The right hindlimbs were used as resting controls. Briefly, mice were anesthetized by intraperitoneal administration using a mix of Domitor^®^ (Orion Pharma Animal Health), Midazolam (Sandoz), and Vetorphale^®^ (Meiji Seika Pharma) at 10 µL/mg body weight and conditioned in the supine position. The left hindlimbs were fixed to a foot-holder connected to an isometric transducer, and the muscle was stimulated at 100 Hz, 80 Hz, 60 Hz, 40 Hz, 20 Hz, 10 Hz, and 1 Hz for 1.5 s each with 1 min interval from each frequency. Five minutes after the last frequency, the GAS muscles were stimulated at 70 Hz for 0.35 s repeated at an interval of 9 s for the first 5 min and the interval was reduced every 5 min to 8, 7, 6, 5, 4, and 3 s. Force was recorded and analyzed using LabChart software (version 7, ADInstrument, Japan). Both exercised and resting muscles were harvested immediately after the fatigue test for biochemical analyses.

### Serum urea nitrogen (BUN) and serum creatinine

Blood was collected and the plasma was immediately separated by centrifugation (10 min at 900×*g*) and stored at − 30 °C. BUN and serum creatinine levels were measured using AU480 analyzer (Beckman Coulter, Krefeld, Germany), which is an instrument for turbidimetric, spectrophotometric and ion-selective electrode measurements.

### Muscle glycogen

Excised GAS muscles were separated into deep and superficial region visually by color. Approximately 10 mg of superficial region was obtained and lysed in 2 N HCl at 100 °C for 2 h and then neutralized with 2 N NaOH. Samples were centrifuged and supernatants were used for measurement. The assay mixture was composed of 50 mM Tris–HCl (pH 8.1), 1 mM MgCl_2_, 0.5 mM ATP, 0.5 mM NADP, 0.14 U/ml glucose-6-phosphate dehydrogenase, and 0.28 U/ml hexokinase. Absorbances were measured on 340 nm wavelength.

### Immunohistochemistry

TA muscles were frozen with optimum cutting temperature embedding compound (OCT) in liquid nitrogen-cooled isopentane. These frozen sections were fixed in 4% paraformaldehyde for 10 min at room temperature (RT, 25 ± 5 °C), then incubated with blocking buffer containing 5% fetal bovine serum, 5% normal goat serum, 2% BSA, 0.2% TritonX-100, and 0.1% sodium azide for 90 min at RT. Sections were subsequently incubated with anti-CD31 (1:200; Abcam ab28364) overnight at 4 °C, followed by secondary antibodies incubation with goat anti-rabbit IgG Alexa Fluor^®^ 594 (1:500; Invitrogen A-11012) at RT for 1 h. Afterwards, sections were counterstained with DAPI (1:2000) for 10 min at RT. All images were captured by Olympus BX53 microscope (Olympus, Tokyo, Japan) and analyzed by Nikon Elements imaging software (Nikon, Tokyo, Japan).

### DNA microarray

Total RNA from GAS muscles was isolated using RNeasy Lipid Tissue Mini Kit, and subjected to cRNA synthesis for a DNA microarray analysis according to the manufacturer’s instructions (44 K whole mouse genome 60-mer oligo microarray, Agilent Technologies, Palo Alto, CA). Fluorescence labeling, hybridization, and image processing were performed according to the manufacturer’s instructions (Agilent Technologies). Briefly, cRNA samples were fragmented and hybridized on the 44 K whole mouse genome oligo microarray slides at 65 °C for 17 h. The glass slides were then washed, and scanned using Agilent DNA microarray scanner (Agilent Technologies). In this experiment, DyeSwap method was carried out in order to eliminate the bias between dyes because the difference between Cyanine 3-CTP (Cy-3) and Cyanine 5-CTP (Cy-5) was altered the efficiency of cRNA hybridization in case of competitive DyeCoupling assay. Gene expression data were obtained using Agilent Feature Extraction software, using defaults for all parameters except ratio terms, which were changed according to the Agilent protocol to fit the direct labeling procedure. Files and images, including error values and *p* values, were obtained by the Agilent Feature Extraction Program (version 9.5).

### Gene functional analysis

To organize functionally related genes into biologically meaningful modules, we use Gene Ontology Encrichment Analysis for efficient interpretation [31–33]. Twenty highest modules with fold enrichment > 1.90 were selected. We ensured no duplication of the official gene symbols were input into the analysis.

### Quantitative PCR analysis

Total RNA was extracted using QIAzol and purified with a RNeasy Lipid Tissue Mini Kit (Qiagen Sciences, Germantown, MD, USA). Reverse transcription was conducted with ReverTra Ace™ (TOYOBO, Osaka, Japan), random primers (TOYOBO), and dNTPs (TOYOBO). For quantitative PCR analysis, cDNA and primers were added to the THUNDERBIRD SYBR qPCR Mix (TOYOBO), to give a total reaction volume of 20 µl. PCR reactions were then performed using StepOnePlusTM (Applied Biosystems, Foster City, CA). The primers used can be found in supplementary data (Table S1).

### Oxidative stress analysis

Oxidative stress in the muscle was assessed by measuring MDA tissue level. Briefly, approximately 25 mg GAS muscle was homogenized in ~ 250 µL RIPA buffer containing protease inhibitors (200 µM PMSF, 10 µg/ml leupeptin, and 10 µg/ml aprotinin). Homogenates were centrifuged at 1600×*g* for 10 min at 4 °C and supernatants were used for measurement using Thiobarbituric Acid Reactive Substances (TBARS) Assay Kit (Cayman, Item No. 10009055).

### Citrate synthase activity

Citrate synthase activity was assessed according to Srere [34] with slight modification. Briefly, GAS muscle was homogenized in a buffer (1:9 mass:volume) containing 175 mM KCl, 10 mM GSH, and 2 mM EDTA (pH 7.4). Homogenates were freeze-thawed 3 times and diluted 50 times in 100 mM Tris/HCl (pH 8.0). Twenty microliters of sample were place into 96-well microplate reader and quickly added with 180 µL assay mixture containing 60 mM Tris/HCl (pH 8.0), 0.3 mM acetyl CoA, 0.1 mM 5,5ʹ-dithiobis (2-nitrobenzonic acid), and 0.5 mM oxalacetic acid. Absorbance was measured every 30 s for 240 s at 412 nm. The slope between 90 and 150 s was divided by the sample weight to calculate the enzyme activity. Measurements were done in triplicate.

### Statistical analysis

Values are expressed as the mean ± S.E. For contraction force and fatigability tests, two-way ANOVA was done to determine the significant difference between control and treatment groups. Student’s *t*-test was used for two groups comparison for the rest of the data. *p* value < 0.05 are considered statistically significant and all statistical analyses were performed using GraphPad Prism 7 software.

## Results

### Body weights and fasting blood glucose levels in STZ models

**Fig. 1:**
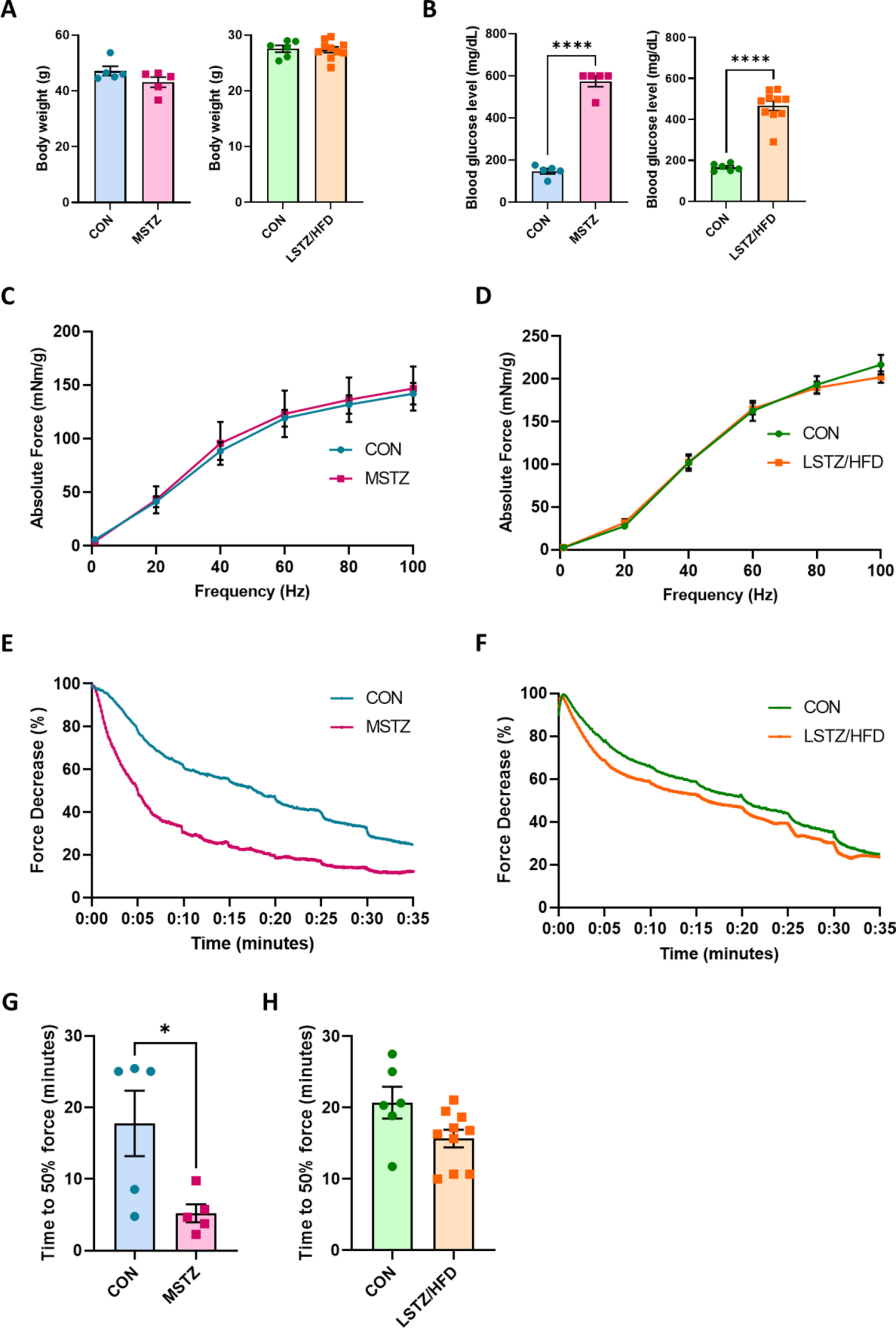
MSTZ model shows more severe phenotype in fatigability test than LSTZ/HFD compared to their respective controls. **A** Final body weight of MSTZ and LSTZ/HFD models at the end of experiment (4 and 8 weeks respectively). **B** Final blood glucose level of MSTZ and LSTZ/HFD models at the end of experiment. **C** Contraction force of MSTZ model (n = 5). **D** Contraction force of LSTZ/HFD model (n = 6–8). **E** Fatigability test of MSTZ model (n = 5). **F** Fatigability test of LSTZ/HFD model (n = 6–8). **G** Time to reach 50% force depression in MSTZ model. **H** Time to reach 50% force depression in LSTZ/HFD model. Values are means ± SEM. Statistical analysis was performed with Student’s t test. *p < 0.05; ****p < 0.0001

Neither STZ model showed any significant difference in body weight compared to their respective controls at 4 and 8 weeks post-STZ injection for the MSTZ and LSTZ/HFD models, respectively (Fig. 1A). Final fasting blood glucose levels were significantly elevated in both models (Fig. 1B), with the MSTZ model showing higher level (574.6 mg/dl) than the LSTZ/HFD (466.7 mg/dl), despite the shorter period. This indicated that the MSTZ model had a more severe effect on insulin secretion, leading to more severe hyperglycemia, than the LSTZ/HFD. Our results are consistent with those of previous studies showing that STZ toxicity in pancreatic beta cells is dose-dependent [35].

### Contraction force was similar, but fatigue resistance differed between MSTZ and STZ/HFD models

Next, we examined the contractile force and fatigue resistance to assess muscle function in both models. The contractile force did not differ between the models and their respective controls (Fig. 1C and D). However, in the fatigability test, the MSTZ group surprisingly showed a more severe phenotype than the LSTZ/HFD model compared to their controls (Fig. 1E and F). Furthermore, the time needed to reach a 50% force-decrease was significantly lower in the MSTZ model, which further emphasizes the more severe outcome of muscle fatigability in the MSTZ model than in the LSTZ/HFD (Fig. 1G and H). To remove the possibility that these results were affected by a different renal impairment in the models, we measured serum creatinine and urea nitrogen (BUN) and both models showed no significant difference compared to their respective controls (Fig. S2A–D). We also examined the state of oxidative stress by measuring malondialdehyde (MDA) level in the skeletal muscle but both models did not significantly differ compared to their controls (Fig. S2E and F).

### Muscle fatigability signatures in MSTZ model

**Fig. 2:**
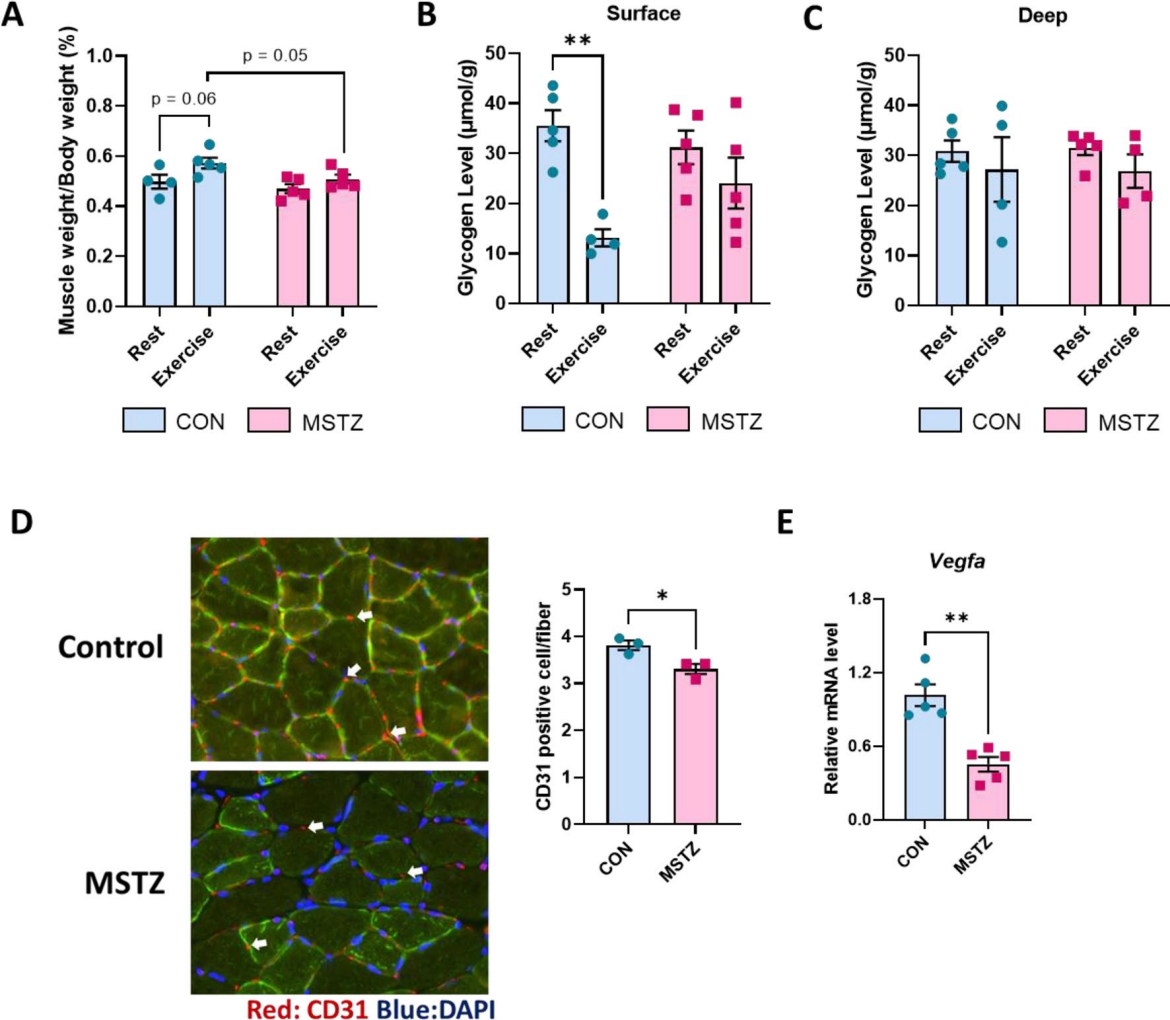
MSTZ model exhibits disrupted glycogen metabolism and reduced vascularization. **A** Muscle weight corrected for body weight pre- and post-exercise in MSTZ model. **B** Glycogen level in surface area of skeletal muscle pre- and post-exercise in MSTZ model. **C** Glycogen level in deep area of skeletal muscle pre- and post-exercise in MSTZ model. **D** CD31 staining for blood vessels in skeletal muscle of MSTZ model. **E** *Vegfa* mRNA expression in MSTZ model. Values are means ± SEM. Statistical analysis was performed with Student’s t test. *p < 0.05; **p < 0.01

To investigate the possible mechanism underlying the more severe decrease in fatigability in the MSTZ model, we checked the change in muscle weight post-exercise. Exercise induces a sharp increase in osmotically active metabolites owing to increased energy metabolism. This leads to cell swelling as water enters the cell to maintain the osmotic balance [36]. However, the MSTZ model showed no significant increase in muscle weight in the exercised muscle compared to the contralateral resting muscle (Fig. 2A). This indicated disrupted energy metabolism within the skeletal muscles of the MSTZ model. Glycogen is an important energy substrate in exercising muscles and decreases after exercise [37]. Thus, we measured the glycogen levels in the resting and exercised muscles of the MSTZ model to confirm the state of energy metabolism. Remarkably, no significant decrease in glycogen content was observed in the MSTZ muscles post-exercise compared to the resting muscle (Fig. 2B and C), suggesting abnormal glycogen utilization in the MSTZ model.

Energy metabolism during exercise, particularly endurance exercise, is tightly associated with oxygen supply to the muscle [38]. To clarify the possibility that disrupted oxygen supply is involved in the severe fatigue observed in MSTZ model, we examined the capillaries in the muscle and found a significantly lower capillary number per fiber in the MSTZ muscles (Fig. 2D). Furthermore, the mRNA expression of vascular endothelial growth factor alpha (*Vegfa*), which plays a major role in angiogenesis [39], was significantly decreased in the MSTZ muscles (Fig. 2E). These results suggest that the lower fatigue resistance in the MSTZ model may be attributed to abnormal glycogen utilization and hypoxia due to decreased vascularization in the MSTZ muscles.

### Muscle fatigability-related genes were differentially expressed in MSTZ model only

**Fig. 3:**
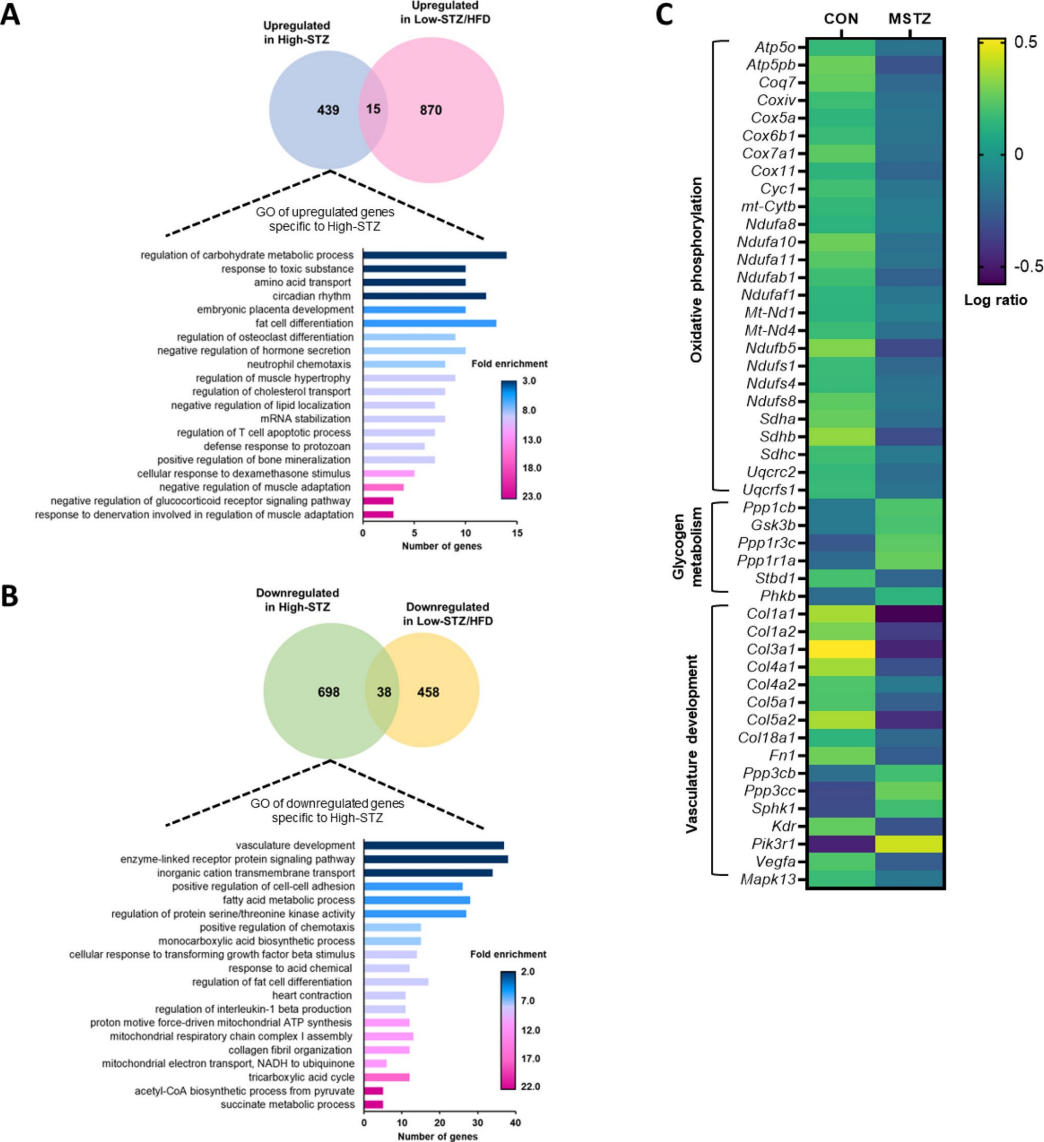
Muscle fatigue-related molecular cues specific to MSTZ model. **A** Molecular signatures of upregulated genes specific to MSTZ model. **B** Molecular signatures of downregulated genes specific to MSTZ model. **C** Selected genes expression related to muscle fatigue specific to MSTZ model

To further understand the mechanism underlying the difference in fatigability results, we conducted gene expression analysis using a DNA microarray. We found 2465 differently expressed genes between the models, with 15 upregulated and 38 downregulated genes in both models. Among these differentially expressed genes, we performed a functional analysis of 439 upregulated and 698 downregulated genes exclusive to the MSTZ model. The upregulated genes indicated enrichment of genes associated with glucocorticoid signaling, carbohydrate metabolism, and the immune system (Fig. 3A). Notably, the downregulated genes highlighted gene enrichment associated with aerobic metabolism and the development of blood vessels (Fig. 3B), which is consistent with the results on capillary number. We also identified 48 genes possibly involved in muscle fatigue mechanism, which belong to ‘oxidative phosphorylation’, and ‘vasculature development’, which are downregulated, and ‘glycogen metabolism’, which is upregulated (Fig. 3C).

**Fig. 4:**
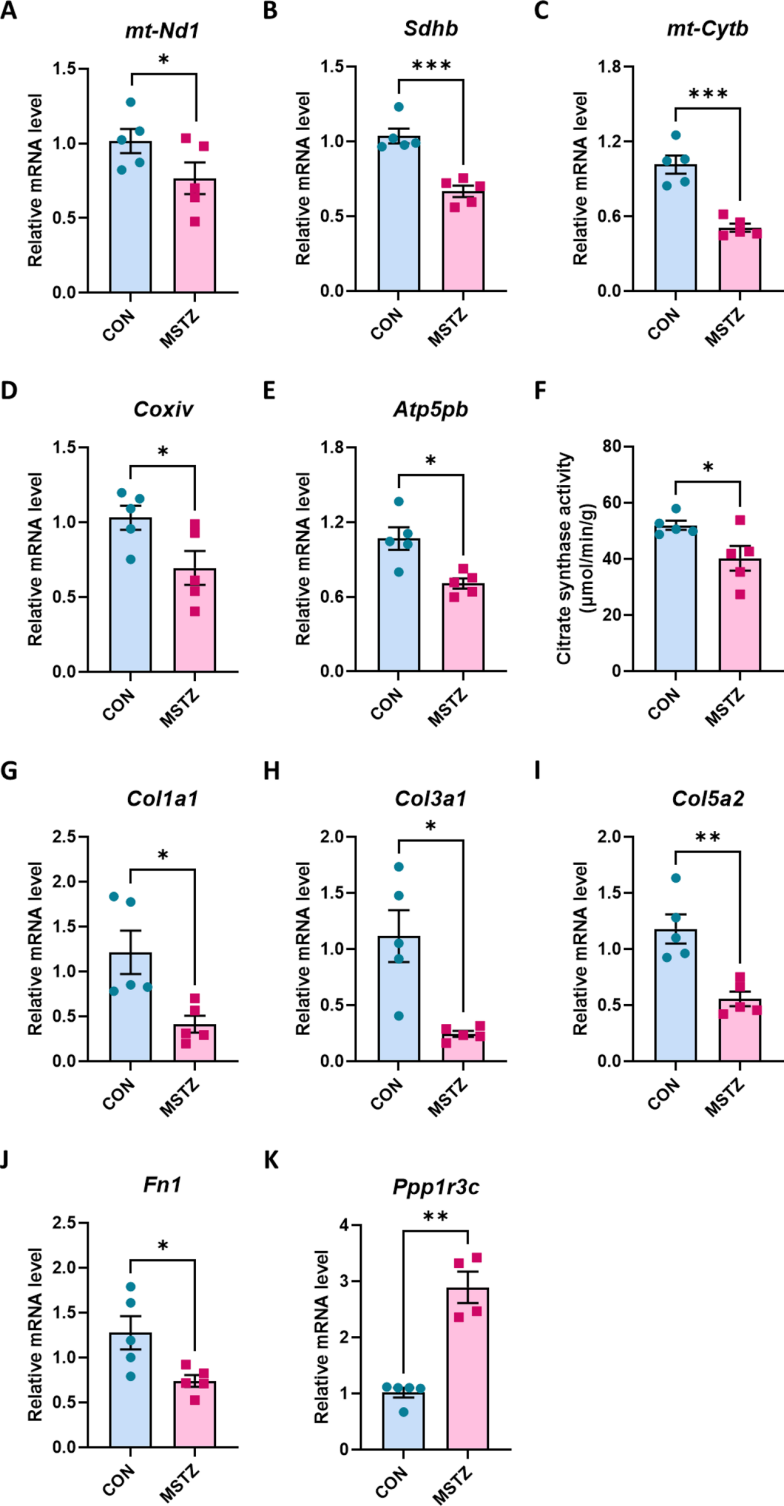
Oxidative phosphorylation- and vasculature development-related genes are significantly downregulated in skeletal muscle of MSTZ model. **A**–**E** Relative mRNA expression of oxidative phosphorylation-related genes in MSTZ model. **F** Citrate synthase activity in the skeletal muscle of MSTZ mode. **G**–**J** Relative mRNA expression of vasculature development-related genes in skeletal muscle of MSTZ model. **K** Relative mRNA expression of glycogen metabolism-related gene, *Ppp1r3c*, in skeletal muscle of MSTZ model. Values are means ± SEM. Statistical analysis was performed with Student’s t test. *p < 0.05; **p < 0.01; ***p < 0.001

As the DNA microarray data revealed that the downregulation of oxidative phosphorylation-related genes was exclusive to the MSTZ model, we performed qPCR analysis to validate the mRNA levels of few of these genes in the MSTZ model. We found significantly downregulated mRNA levels of genes belonging to the electron transport chain, such as *mt-Nd1* (complex I), *Sdhb* (complex II), *mt-Cytb* (complex III), and *Coxiv* (complex IV), and mitochondrial coupling genes, such as *Atp5pb* (ATP synthase) (Fig. 4A–E). To clarify whether these altered gene expressions are reflected to the mitochondrial function, we measured citrate synthase activity, the main rate limiting enzyme in TCA cycle and has been used as a marker for mitochondrial density and function [40–42]. Our data showed that citrate synthase activity was significantly lower in MSTZ model (Fig. 4F), which was not observed in LSTZ/HFD model (Fig. S2G). We also confirmed the downregulation of vascularization-related genes such as *Fn1*, *Col1a1*, *Col5a2*, and *Col3a1* (Fig. 4G–J). *Ppp1r3c* gene, which is involved in glycogen metabolism, was significantly upregulated in MSTZ model (Fig. 4K). Overall, these results support the view that the MSTZ model exhibits more severe fatigue owing to the disrupted glycogen and aerobic metabolism associated with decreased vascularization.

## Discussion

STZ is commonly used to induce diabetes in animal models and has been extensively adopted to study diabetes and its complications [8]. However, concerns have arisen as studies have reported nonspecific toxicity to tissues other than beta cells, such as the kidneys, especially with high-dose administration [9, 10]. To address these concerns, two models employing moderate-dose STZ and a combination of low-dose STZ and an HFD have been developed [14]. Both models are arbitrarily used in studies on diabetic complications; however, no study has evaluated whether these models affect muscle function similarly. Our study showed that moderate-dose STZ and low-dose STZ/HFD produced similar results in muscle contraction force, but differed in fatigue resistance.

Previous studies on contraction force in STZ-induced diabetic animals are unclear, with the majority showing a decrease [5, 7, 22–24], while some showed no change [5, 24–26] or an increase [6, 27, 28]. However, studies showing decreased force employed high-dose STZ; therefore, it is unclear if the results were due to direct STZ toxicity in muscles or secondary to hyperglycemia/hypoinsulinemia. Furthermore, as STZ can directly affect the kidneys via the GLUT2 transporter [11], altered muscle function associated with kidney dysfunction cannot be excluded. Our results indicated that in models using moderate and low STZ doses, the contraction force remained unchanged despite established hyperglycemia. Johnston et al. showed that high-dose STZ directly affects myoblast and muscle growth in young rats [43]. Further study comparing contraction force in STZ-induced mice (120 mg/kg) and *Ins2*^*Akita*+/−^ showed that no force loss was observed in *Ins2*^*Akita*+*/−*^; however, a significant increase in contraction force was observed in STZ-induced mice [28]. Another study employing *db/db* mice also reported no differences in contraction force [44]. These results suggest that moderate-dose STZ and low-dose STZ/HFD produce similar effects on contraction force compared to non-STZ diabetic models. We cannot disregard the possibility that the current models have not yet reached the severity at which the contraction force is affected, as studies have shown that the reduced contractile force in STZ-induced animals depends on the severity of the diabetic state [5, 22, 23]. Nevertheless, these models could be better options for examining the contraction force in diabetic muscles, and the results obtained from high-dose STZ models should be interpreted with caution.

Notably, our findings revealed that only the MSTZ model showed a severe decrease in fatigue resistance, likely due to the lack of oxygen supply via reduced capillarity, which led to altered glycogen metabolism and oxidative phosphorylation. Poor fatigue resistance in individuals with diabetes has been frequently reported and has become a hurdle in disease management [45–47]. During endurance exercise, muscles rely on anaerobic energy for the initial 1–2 min, followed by aerobic metabolism, which subsequently dominates ATP synthesis for the remainder of the exercise [38]. Hence, angiogenesis is crucial for ensuring sufficient oxygen supply for ATP synthesis during exercise. VEGF is a key player in exercise-induced angiogenesis and its deficiency has been associated with poor endurance [39]. Consistent with our data, VEGFa downregulation has been reported in the muscles of individuals with diabetes and is correlated with exercise capacity [4]. Additionally, we found no difference in glycogen levels between the resting and exercised muscles, suggesting disrupted glycogen metabolism. Glycogen is an important substrate for the initial energy supply during exercise, and its altered metabolism affects exercise performance [37]. Among the genes upregulated only in the MSTZ model, protein phosphatase 1, regulatory subunit 3C (*Ppp1r3c*), which encodes PPP1R3C, also known as PTG, was upregulated in the MSTZ muscle. PTG directly binds to protein phosphatase (PP1) under hypoxic conditions in a hypoxia-inducible factor (HIF)-dependent manner. This complex causes glycogen accumulation via its action on glycogen phosphorylase (GP) [48]. This suggests that reduced oxygen supply increased *Ppp1r3c* in the MSTZ model, which plays a role in repressing glycogen breakdown through the formation of the PTG–PP1 complex and its regulation of GP, and possibly affects fatigue resistance. Furthermore, our data demonstrated that genes associated with oxidative phosphorylation, including *mt-Cytb, Sdhb, Coxiv, mt-Nd1* (electron transport chains), and *Atp5pb* (mitochondrial coupling), were downregulated in the MSTZ model. Altered electron transport chain flux and mitochondrial uncoupling, which impact exercise performance, have been frequently reported in aging muscles [49]. Lower citrate synthase activity, which has been observed in aging muscles [50], further emphasized mitochondrial dysfunction in this model. Hence, blunted mitochondrial function may also be involved in the severe fatigue observed in the MSTZ model. Notably, since these phenotypes and molecular cues were exclusive to the MSTZ model, the use of a low-STZ/HFD in examining the fatigue resistance of diabetic muscles should be cautiously considered.

The pathological relationship between chronic kidney disease and the skeletal muscle is currently a popular research topic. Our previous report revealed that renal inflammation was observed in the MSTZ model independent of STZ toxicity [51], suggesting a possible interplay between kidney dysfunction and skeletal muscle function in this model. For instance, increased glucocorticoid levels are a pathological response to kidney injury and have been reported to reduce angiogenesis in skeletal muscle [52, 53]. Further investigations of the possible crosstalk between the kidneys and skeletal muscles are needed to explain the pathological differences between these two models. Overall, our findings demonstrate that moderate-dose STZ and the combination of low-dose STZ with HFD yield similar outcomes in muscle contraction yet differ in fatigue resistance. Although both models may be used interchangeably to assess muscle contraction in diabetic myopathy, careful consideration of the molecular signatures of each model is necessary to select a suitable model for fatigability assessment. We further show that moderate-dose STZ exhibits decreased capillarization and mitochondrial dysfunction. These results offer the possibility of targeting mitochondrial activation to combat diabetic myopathy and overall diabetic states. For instance, previous studies show that caloric restriction, exercise, and several drugs (resveratrol, AICAR, and bezafibrate) can induce mitochondrial biogenesis via PGC-1α activation [54, 55]. Employing these treatments in therapeutic strategies of diabetes may be beneficial for improving muscle function and overall conditions of diabetic patients. Future investigation on this possibility is warranted.

## Supplementary Information

Below is the link to the electronic supplementary material.

Supplementary file1 (DOCX 111 KB)

## References

[1] Lin X, Xu Y, Pan X et al (2020) Global, regional, and national burden and trend of diabetes in 195 countries and territories: an analysis from 1990 to 2025. Sci Rep 10(1):14790. 10.1038/s41598-020-71908-9 32901098 10.1038/s41598-020-71908-9 PMC7478957

[2] D’Souza DM, Al-Sajee D, Hawke TJ (2013) Diabetic myopathy: impact of diabetes mellitus on skeletal muscle progenitor cells. Front Physiol 4:379. 10.3389/fphys.2013.00379 24391596 10.3389/fphys.2013.00379 PMC3868943

[3] Morley JE (2008) Diabetes, sarcopenia, and frailty. Clin Geriatr Med 24(3):455–469. 10.1016/j.cger.2008.03.004 18672182 10.1016/j.cger.2008.03.004

[4] Kivelä R, Silvennoinen M, Touvra A et al (2006) Effects of experimental type 1 diabetes and exercise training on angiogenic gene expression and capillarization in skeletal muscle. FASEB J 20(9):1570–1572. 10.1096/fj.05-4780fje 16816123 10.1096/fj.05-4780fje

[5] Stephenson GMM, O’Callaghan A, Stephenson DG (1994) Single-fiber study of contractile and biochemical properties of skeletal muscles in streptozotocin-induced diabetic rats. Diabetes 43(5):622–628. 10.2337/diab.43.5.622 8168636 10.2337/diab.43.5.622

[6] Vignaud A, Ramond F, Hourdé C, Keller A, Butler-Browne G, Ferry A (2007) Diabetes provides an unfavorable environment for muscle mass and function after muscle injury in mice. Pathobiology 74(5):291–300. 10.1159/000105812 17890896 10.1159/000105812

[7] Fortes MAS, Pinheiro CHJ, Guimarães-Ferreira L, Vitzel KF, Vasconcelos DAA, Curi R (2015) Overload-induced skeletal muscle hypertrophy is not impaired in STZ-diabetic rats. Physiol Rep 3(7):e12457. 10.14814/phy2.12457 26197932 10.14814/phy2.12457 PMC4552534

[8] Furman BL (2021) Streptozotocin-induced diabetic models in mice and rats. Curr Protoc. 10.1002/cpz1.78 33905609 10.1002/cpz1.78

[9] Mohammed-Ali Z, Carlisle RE, Nademi S, Dickhout JG (2017) Animal models of kidney disease. In: Michael Conn P (ed) Animal models for the study of human disease. Elsevier, Amsterdam, pp 379–417

[10] Breyer MD, Bottinger E, Brosius F et al (2005) Mouse models of diabetic nephropathy. J Am Soc Nephrol 16(1):27–45. 10.1681/ASN.2004080648 15563560 10.1681/ASN.2004080648

[11] Brouwers B, Pruniau V, Cauwelier E et al (2013) Phlorizin pretreatment reduces acute renal toxicity in a mouse model for diabetic nephropathy. J Biol Chem 288(38):27200–27207. 10.1074/jbc.M113.469486 23940028 10.1074/jbc.M113.469486 PMC3779717

[12] Zhang M, Lu XY, Li J, Xu ZG, Chen L (2008) The characterization of high-fat diet and multiple low-dose streptozotocin induced type 2 diabetes rat model. Exp Diabetes Res 2008:1–9. 10.1155/2008/704045 10.1155/2008/704045 PMC2613511 19132099

[13] Chao PC, Li Y, Chang CH, Shieh JP, Cheng JT, Cheng KC (2018) Investigation of insulin resistance in the popularly used four rat models of type-2 diabetes. Biomed Pharmacother 101:155–161. 10.1016/j.biopha.2018.02.084 29486333 10.1016/j.biopha.2018.02.084

[14] Tesch GH, Allen TJ (2007) Rodent models of streptozotocin-induced diabetic nephropathy (Methods in Renal Research). Nephrology 12(3):261–266. 10.1111/j.1440-1797.2007.00796.x 17498121 10.1111/j.1440-1797.2007.00796.x

[15] Martinez-Huenchullan SF, McLennan SV, Ban LA, Morsch M, Twigg SM, Tam CS (2017) Utility and reliability of non-invasive muscle function tests in high-fat-fed mice. Exp Physiol 102(7):773–778. 10.1113/EP086328 28497900 10.1113/EP086328

[16] Iyer SR, Valencia AP, Hernández-Ochoa EO, Lovering RM (2016) In vivo assessment of muscle contractility in animal studies. Methods in molecular biology, vol 1460. Humana Press Inc, New Jersey, pp 293–307 10.1007/978-1-4939-3810-0_20 PMC5500964 27492180

[17] Manabe Y, Fujii NL (2016) Experimental research models for skeletal muscle contraction. J Phys Fit Sports Med 5(5):373–377. 10.7600/jpfsm.5.373

[18] Watanabe D, Aibara C, Wada M (2019) Treatment with EUK-134 improves sarcoplasmic reticulum Ca^2+^ release but not myofibrillar Ca^2+^ sensitivity after fatiguing contraction of rat fast-twitch muscle. Am J Physiol Regul Integr Comp Physio 316(5):R543–R551. 10.1152/ajpregu.00387.2018 10.1152/ajpregu.00387.2018 30794441

[19] Aibara C, Okada N, Watanabe D, Shi J, Wada M (2020) Effects of high-intensity interval exercise on muscle fatigue and SR function in rats: a comparison with moderate-intensity continuous exercise. J Appl Physiol 129(2):343–352. 10.1152/japplphysiol.00223.2020 32673156 10.1152/japplphysiol.00223.2020

[20] Balch MHH, Harris H, Chugh D et al (2021) Ischemic stroke-induced polyaxonal innervation at the neuromuscular junction is attenuated by robot-assisted mechanical therapy. Exp Neurol 343:113767. 10.1016/j.expneurol.2021.113767 34044000 10.1016/j.expneurol.2021.113767 PMC8286354

[21] Wang Q, Hernández-Ochoa E, Viswanathan MC et al (2021) CaMKII oxidation is a critical performance/disease trade-off acquired at the dawn of vertebrate evolution. Nat Commun 12(1):3175. 10.1038/s41467-021-23549-3 34039988 10.1038/s41467-021-23549-3 PMC8155201

[22] Cotter M, Cameron NE, Lean DR, Robertson S (1989) Effects of long-term streptozotocin diabetes on the contractile and histochemical properties of rat muscles. Q J Exp Physiol 74(1):65–74. 10.1113/expphysiol.1989.sp003240 2524084 10.1113/expphysiol.1989.sp003240

[23] Fahim MA, El-Sabban F, Davidson N (1998) Muscle contractility decrement and correlated morphology during the pathogenesis of streptozotocin-diabetic mice. Anat Rec 251(2):240–244. 10.1002/(SICI)1097-0185(199806)251:2%3c240::AID-AR13%3e3.0.CO;2-O 9624455 10.1002/(SICI)1097-0185(199806)251:2<240::AID-AR13>3.0.CO;2-O

[24] Sanchez OA, Snow LM, Lowe DA, Serfass RC, Thompson LV (2005) Effects of endurance exercise-training on single-fiber contractile properties of insulin-treated streptozotocin-induced diabetic rats. J Appl Physiol 99(2):472–478. 10.1152/japplphysiol.01233.2004 15831797 10.1152/japplphysiol.01233.2004

[25] McGuire M, MacDermott M (1999) The influence of streptozotocin diabetes and metformin on erythrocyte volume and on the membrane potential and the contractile characteristics of the extensor digitorum longus and soleus muscles in rats. Exp Physiol 84(6):1051–1058. 10.1111/j.1469-445X.1999.01916.x 10564702

[26] Lesniewski LA, Miller TA, Armstrong RB (2003) Mechanisms of force loss in diabetic mouse skeletal muscle. Muscle Nerve 28(4):493–500. 10.1002/mus.10468 14506722 10.1002/mus.10468

[27] McGuire M, Dumbleton M, MacDermott M, Bradford A (2001) Contractile and electrical properties of sternohyoid muscle in streptozotocin diabetic rats. Clin Exp Pharmacol Physiol 28(3):184–187. 10.1046/j.1440-1681.2001.03433.x 11207673 10.1046/j.1440-1681.2001.03433.x

[28] Krause MP, Riddell MC, Gordon CS, Imam SA, Cafarelli E, Hawke TJ (2008) Diabetic myopathy differs between Ins2^Akita+/−^ and streptozotocin-induced type 1 diabetic models. J Appl Physiol 106(5):1650–1659. 10.1152/japplphysiol.91565.2008 10.1152/japplphysiol.91565.2008 19246652

[29] Like AA, Rossini AA (1976) Streptozotocin-induced pancreatic insulitis: new model of diabetes mellitus. Science 193(4251):415–417. 10.1126/science.180605 180605 10.1126/science.180605

[30] Schlöndorff D (2010) Choosing the right mouse model for diabetic nephropathy. Kidney Int 77(9):749–750. 10.1038/ki.2009.545 20393486 10.1038/ki.2009.545

[31] Aleksander SA, Balhoff J, Carbon S et al (2023) The gene ontology knowledgebase in 2023. Genetics. 10.1093/genetics/iyad031 36866529 10.1093/genetics/iyad031 PMC10158837

[32] Ashburner M, Ball C, Blake J et al (2000) Gene Ontology: tool for the unification of biology. Nat Genet 25(1):25–29. 10.1038/75556 10802651 10.1038/75556 PMC3037419

[33] Thomas PD, Ebert D, Muruganujan A, Mushayahama T, Albou L, Mi H (2022) PANTHER: making genome-scale phylogenetics accessible to all. Protein Sci 31(1):8–22. 10.1002/pro.4218 34717010 10.1002/pro.4218 PMC8740835

[34] Srere PA (1969) Citrate synthase. Methods enzymol, vol 13. Academic Press, Cambridge, pp 3–5

[35] Lu WT, Juang JH, Hsu BTS, Huang HS (1998) Effects of high or low dose of streptozotocin on pancreatic islets in C57BL/6 and C.B17-SCID mice. Transplant Proc 30(2):609–610. 10.1016/S0041-1345(97)01425-5 9532197 10.1016/s0041-1345(97)01425-5

[36] Lindinger MI (2022) A century of exercise physiology: key concepts in muscle cell volume regulation. Eur J Appl Physiol 122(3):541–559. 10.1007/s00421-021-04863-6 35037123 10.1007/s00421-021-04863-6

[37] Jensen TE, Richter EA (2012) Regulation of glucose and glycogen metabolism during and after exercise. J Physiol 590(5):1069–1076. 10.1113/jphysiol.2011.224972 22199166 10.1113/jphysiol.2011.224972 PMC3381815

[38] Hargreaves M, Spriet LL (2020) Skeletal muscle energy metabolism during exercise. Nat Metab 2(9):817–828. 10.1038/s42255-020-0251-4 32747792 10.1038/s42255-020-0251-4

[39] Wagner PD (2011) The critical role of VEGF in skeletal muscle angiogenesis and blood flow. Biochem Soc Trans 39(6):1556–1559. 10.1042/BST20110646 22103486 10.1042/BST20110646

[40] Yubero D, Adin A, Montero R et al (2016) A statistical algorithm showing coenzyme Q10 and citrate synthase as biomarkers for mitochondrial respiratory chain enzyme activities. Sci Rep 6(1):15. 10.1038/s41598-016-0008-1 28442759 10.1038/s41598-016-0008-1 PMC5431365

[41] Jacobs RA, Diaz V, Meinild A, Gassmann M, Lundby C (2013) The C57Bl/6 mouse serves as a suitable model of human skeletal muscle mitochondrial function. Exp Physiol 98(4):908–921. 10.1113/expphysiol.2012.070037 23180810 10.1113/expphysiol.2012.070037

[42] Larsen S, Nielsen J, Hansen C et al (2012) Biomarkers of mitochondrial content in skeletal muscle of healthy young human subjects. J Physiol 590(14):3349–3360. 10.1113/jphysiol.2012.230185 22586215 10.1113/jphysiol.2012.230185 PMC3459047

[43] Johnston APW, Campbell JE, Found JG, Riddell MC, Hawke TJ (2007) Streptozotocin induces G2 arrest in skeletal muscle myoblasts and impairs muscle growth in vivo. Am J Physiol Cell Physiol 292(3):C1033–C1040. 10.1152/ajpcell.00338.2006 17092995 10.1152/ajpcell.00338.2006

[44] Yamamoto H, Eshima H, Kakehi S, Kawamori R, Watada H, Tamura Y (2022) Impaired fatigue resistance, sarcoplasmic reticulum function, and mitochondrial activity in soleus muscle of db/db mice. Physiol Rep. 10.14814/phy2.15478 36117307 10.14814/phy2.15478 PMC9483406

[45] McDermott A, Nevin A, Gildea N, Rocha J, O’Shea D, Egaña M (2023) Muscle deoxygenation during ramp incremental cycle exercise in older adults with type 2 diabetes. Eur J Appl Physiol. 10.1007/s00421-023-05297-y 37638974 10.1007/s00421-023-05297-y PMC10858067

[46] Kim Y, Seifert T, Brassard P et al (2015) Impaired cerebral blood flow and oxygenation during exercise in type 2 diabetic patients. Physiol Rep 3(6):e12430. 10.14814/phy2.12430 26109188 10.14814/phy2.12430 PMC4510631

[47] Huebschmann AG, Reis E, Emsermann C et al (2009) Women with type 2 diabetes perceive harder effort during exercise than nondiabetic women. Appl Physiol Nutr Metab 34(5):851–857. 10.1139/H09-074 19935846 10.1139/H09-074

[48] Shen GM, Zhang FL, Liu XL, Zhang JW (2010) Hypoxia-inducible factor 1-mediated regulation of PPP1R3C promotes glycogen accumulation in human MCF-7 cells under hypoxia. FEBS Lett 584(20):4366–4372. 10.1016/j.febslet.2010.09.040 20888814 10.1016/j.febslet.2010.09.040

[49] Conley KE (2016) Mitochondria to motion: optimizing oxidative phosphorylation to improve exercise performance. J Exp Biol 219(2):243–249. 10.1242/jeb.126623 26792336 10.1242/jeb.126623 PMC6514472

[50] Yeo D, Kang C, Gomez-Cabrera C, Vina J, Ji LL (2019) Intensified mitophagy in skeletal muscle with aging is downregulated by PGC-1alpha overexpression in vivo. Free Radic Biol Med 130:361–369. 10.1016/j.freeradbiomed.2018.10.456 30395971 10.1016/j.freeradbiomed.2018.10.456

[51] Saliu TP, Yazawa N, Hashimoto K et al (2022) Serum amyloid A3 promoter-driven luciferase activity enables visualization of diabetic kidney disease. Int J Mol Sci 23(2):899. 10.3390/ijms23020899 35055081 10.3390/ijms23020899 PMC8779903

[52] Wang XH, Price SR (2023) Organ crosstalk contributes to muscle wasting in chronic kidney disease. Semin Nephrol 43(2):151409. 10.1016/j.semnephrol.2023.151409 37611335 10.1016/j.semnephrol.2023.151409

[53] Shikatani EA, Trifonova A, Mandel E et al (2012) Inhibition of proliferation, migration and proteolysis contribute to corticosterone-mediated inhibition of angiogenesis. PLoS ONE 7(10):e46625. 10.1371/journal.pone.0046625 23056375 10.1371/journal.pone.0046625 PMC3462789

[54] Komen JC, Thorburn DR (2013) Turn up the power—pharmacological activation of mitochondrial biogenesis in mouse models. Br J Pharmacol 171(8):1818–1836. 10.1111/bph.12413 10.1111/bph.12413 PMC3976607 24102298

[55] Valero T (2014) Mitochondrial biogenesis: pharmacological approaches. Curr Pharm Des 20(35):5507–5509. 10.2174/138161282035140911142118 24606795 10.2174/138161282035140911142118

