# Supplementary material for: Comparative study on muscle function in two different streptozotocin-induced diabetic models
Source: Acta Diabetol. 2024 Jun 10;61(11):1443–53. doi: 10.1007/s00592-024-02311-3 (PMC11531449; doi:10.1007/s00592-024-02311-3)
Supplement: Supplementary file 1 — Supplementary file1 (DOCX 111 KB) [file 592_2024_2311_MOESM1_ESM.docx]

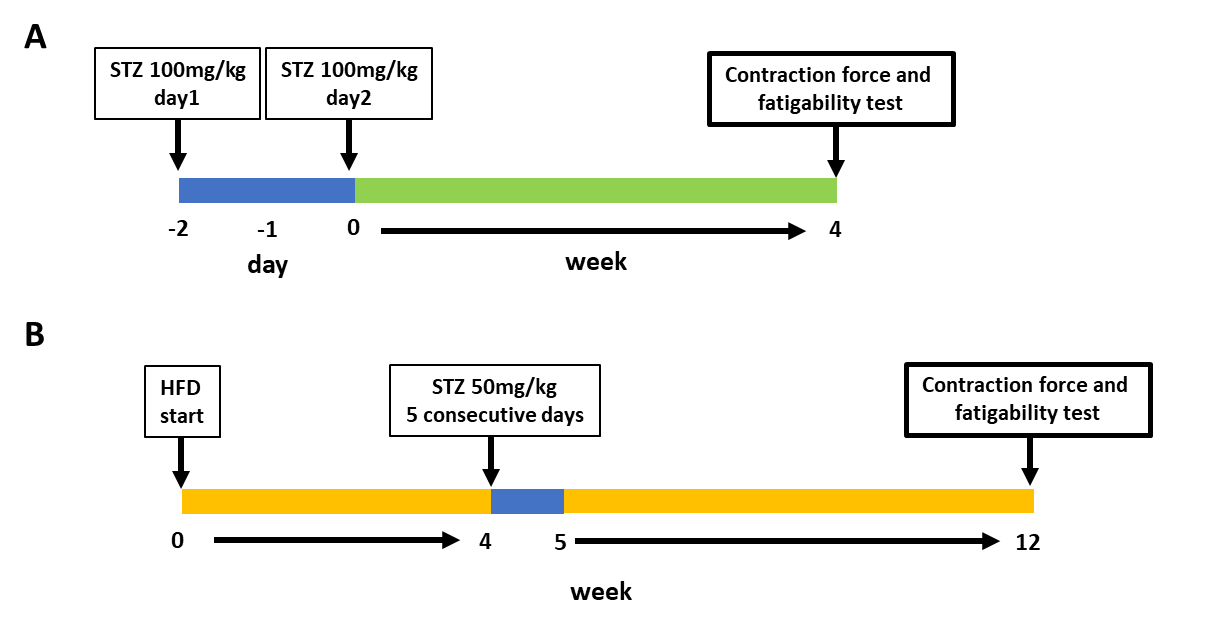


**Figure S1. Experimental design for STZ models**

(A) Moderate-dose STZ model (MSTZ)

(B) Low-dose STZ + high-fat diet model (LSTZ/HFD)


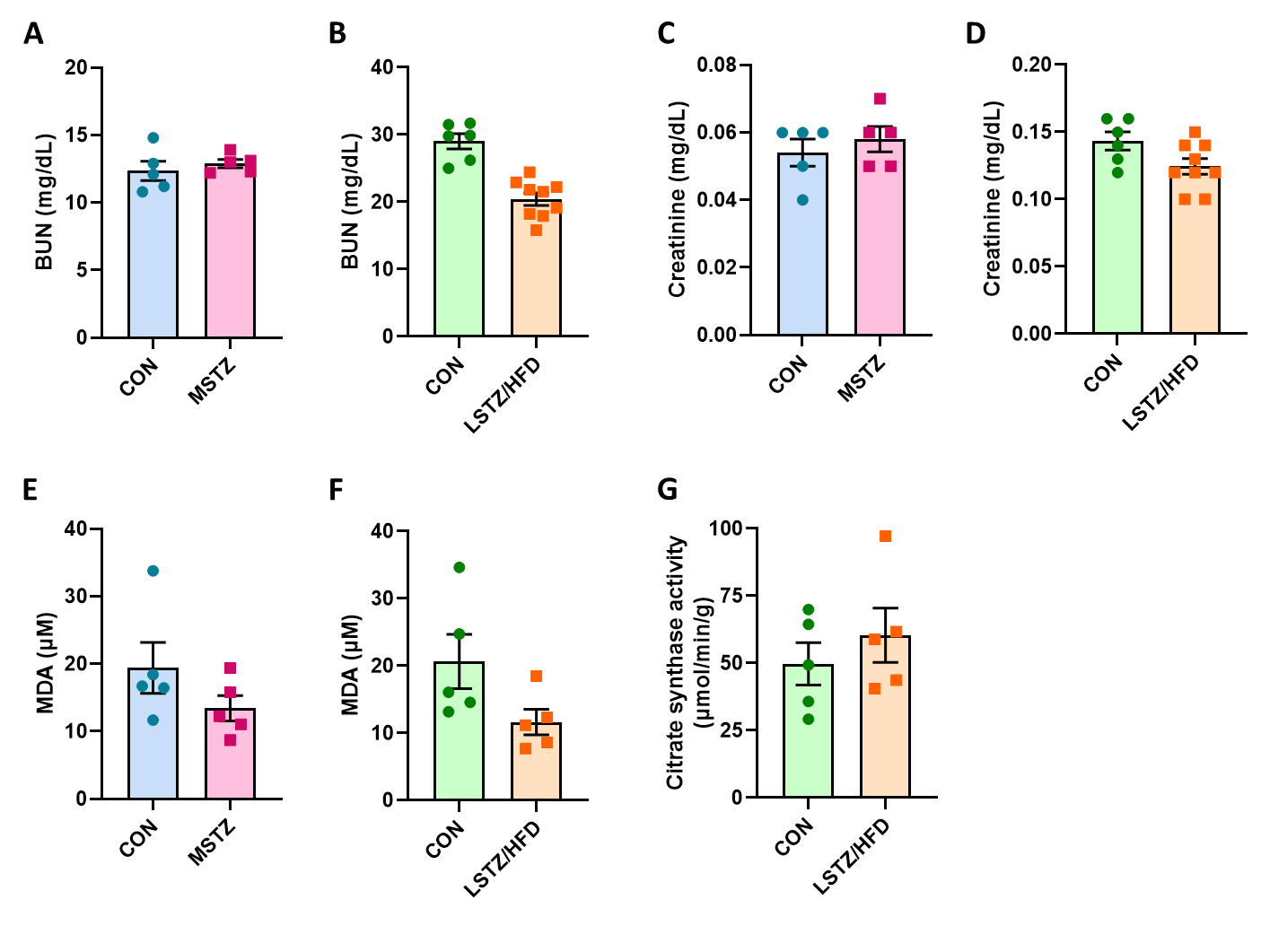


**Figure S2. Biomarkers of renal function, oxidative stress and mitochondrial function**

(A) Serum urea nitrogen (BUN) level in MSTZ model

(B) Serum urea nitrogen (BUN) level in LSTZ/HFD model

(C) Serum creatinine level in MSTZ model

(D) Serum creatinine level in LSTZ/HFD model

(E) Malondialdehyde (MDA) level in skeletal muscle of MSTZ model

(F) MDA levels in skeletal muscle of LSTZ/HFD model

(G) Citrate synthase activity in skeletal muscle of LSTZ/HFD model

Values are means ± SEM.

**Table S1. List of primers used for qPCR analysis**

| **Genes** | **Forward** | **Reverse** |
| --- | --- | --- |
| *Atp5pb* | GTCCAGGGGTATTACAGGCAA | TCAGGAATCAGCCCAAGACG |
| *Col1a1* | CCCAAGGAAAAGAAGCACGTC | ACATTAGGCGCAGGAAGGTCA |
| *Col3a1* | TGGTCCTCAGGGTGTAAAGG | GTCCAGCATCACCTTTTGGT |
| *Col5a2* | TTGGAAACCTTCTCCATGGTCAGA | TCCCCAGTGGGTGTTATAGGA |
| *Coxiv* | CCATTTCTACTTCGGTGTGC | CGTTAAACTGGATGCGGTAC |
| *Fn1* | GGTGACACTTATGAGCGCCCTAAA | AACATGTAACCACCAGTCTCATGTG |
| *mt-Cytb* | ACGTCCTTCCATGAGGACAA | GGAGGTGAACGATTGCTAGG |
| *mt-Nd1* | CAGGATGAGCCTCAAACTCC | CCGGTTTGTTTCTGCTAGGG |
| *Ppp1r3c* | CGGGTCGTGTTTGCGGGACTCC | GGTCACCGTCCGATCTTGCAA |
| *Sdhb* | AATTTGCCATTTACCGATGGGA | AGCATCCAACACCATAGGTCC |
